# Supplementary material for: Environmentally-induced epigenetic conversion of a piRNA cluster
Source: eLife. 2019 Mar 15;8:e39842. doi: 10.7554/eLife.39842 (PMC6420265; doi:10.7554/eLife.39842)
Supplement: Supplementary file 1 — BX2OFF and BX2ON are recombined lines carrying the P(TARGET)GS and the BX2 locus transgenes on the same chromosome. Numbers show the fraction of females harboring complete germline repression of P(TARGET)GS at each generation. Complete stability of the initial epigenetic state was observed at 25°C for BX2OFF and BX2ON lines, 0% repression (n = 415) and 100% repression (n = 339), respectively. At 29°C, all BX2OFF lines showed emergence of silencing capacities, 24.7% (n = 3812). BX2ON lines maintained their silencing capacities over generations at 29°C, 100% (n = 377). nt: not tested. [file elife-39842-supp1.docx]

|  | **25°C** | | | | **29°C** | | | | | | | | | |
| --- | --- | --- | --- | --- | --- | --- | --- | --- | --- | --- | --- | --- | --- | --- |
|  | ***BX2^OfF^*** | | ***BX2^ON^*** | | ***BX2^OfF^*** | | | | | | | | ***BX2^ON^*** | |
| **Lines** | 1 | 2 | 1 | 2 | 1A | 1B | 1C | 1D | 2A | 2B | 2C | 2D | 1 | 2 |
| **G1** | 0/7 | 0/8 | 8/8 | 8/8 | 0/14 | 0/14 | 0/14 | 0/15 | 0/15 | 0/15 | 0/15 | 0/15 | 9/9 | 10/10 |
| **G2** | 0/11 | 0/12 | 12/12 | 12/12 | 0/9 | 0/12 | 0/10 | 0/9 | 0/13 | 0/7 | 0/16 | 1/28 | 12/12 | 9/9 |
| **G3** | 0/12 | 0/3 | 7/7 | 8/8 | 1/17 | 0/14 | 0/16 | 2/14 | 1/18 | 1/23 | 2/23 | 0/23 | 7/7 | 10/10 |
| **G4** | 0/15 | 0/15 | 10/10 | 10/10 | 0/7 | 1/7 | 1/16 | 2/11 | 0/17 | 1/20 | 4/23 | 1/23 | 10/10 | 8/8 |
| **G5** | 0/12 | 0/12 | 12/12 | 12/12 | 2/20 | 6/31 | 3/39 | 11/27 | 0/38 | 0/33 | 0/3 | 0/24 | 8/8 | 6/6 |
| **G6** | 0/6 | 0/8 | 8/8 | 8/8 | 0/2 | 4/20 | 1/8 | 1/18 | 0/9 | 0/15 | 1/14 | 0/4 | 1/1 | 1/1 |
| **G7** | 0/8 | 0/8 | 8/8 | 8/8 | 6/19 | 7/21 | 2/36 | 2/21 | 2/38 | 1/20 | 3/17 | 2/37 | 7/7 | 8/8 |
| **G8** | 0/10 | 0/8 | 6/6 | 8/8 | 3/33 | 11/33 | 0/49 | 8/31 | 5/22 | 14/35 | 5/33 | 6/50 | 8/8 | 8/8 |
| **G9** | nt | nt | nt | nt | 10/23 | 13/21 | 1/22 | 5/28 | 3/26 | 17/44 | 3/36 | 4/38 | 8/8 | 8/8 |
| **G10** | 0/8 | 0/8 | 8/8 | 8/8 | 8/23 | 16/21 | 1/22 | 4/23 | 11/21 | 7/25 | 7/35 | 5/24 | 8/8 | 8/8 |
| **G11** | 0/8 | 0/8 | 8/8 | 8/8 | 7/14 | 16/18 | 1/15 | 3/15 | 1/16 | 8/16 | 2/16 | 4/16 | 8/8 | 8/8 |
| **G12** | 0/8 | 0/8 | 8/8 | 8/8 | 5/14 | 7/8 | 1/5 | 0/8 | 0/6 | 2/17 | 3/18 | 5/21 | 8/8 | 5/5 |
| **G13** | 0/10 | 0/2 | 6/6 | 5/5 | 7/11 | 11/11 | 0/37 | 1/9 | nt | 6/19 | 1/25 | 5/18 | 9/9 | 8/8 |
| **G14** | 0/6 | 0/10 | 5/5 | 5/5 | 28/41 | 25/25 | 0/24 | 5/41 | nt | 6/11 | 0/24 | 7/24 | 6/6 | 5/5 |
| **G15** | 0/12 | 0/4 | 8/8 | 8/8 | 14/22 | 16/16 | 3/21 | 1/23 | nt | 12/23 | 1/24 | 2/20 | 8/8 | 8/8 |
| **G16** | 0/12 | 0/11 | 8/8 | 5/5 | 3/11 | 17/17 | 1/17 | 2/17 | nt | 11/20 | 2/20 | 0/19 | 8/8 | 8/8 |
| **G17** | 0/8 | 0/12 | 5/5 | 7/7 | 5/9 | 22/22 | 4/18 | 0/6 | nt | 9/16 | 0/13 | 0/19 | 8/8 | 3/3 |
| **G18** | 0/11 | 0/12 | 8/8 | 8/8 | 2/8 | 6/6 | 1/10 | 0/5 | nt | 13/21 | 3/8 | 0/14 | 8/8 | 8/8 |
| **G19** | 0/11 | 0/12 | 6/6 | 8/8 | 6/42 | 21/21 | 4/38 | 6/17 | nt | 16/32 | 5/18 | 0/44 | 8/8 | 8/8 |
| **G20** | 0/12 | 0/3 | 6/6 | 6/6 | 4/13 | 21/21 | 4/17 | 10/18 | nt | 10/20 | 12/33 | 0/36 | 8/8 | 8/8 |
| **G21** | 0/12 | 0/12 | 8/8 | 8/8 | 5/16 | 12/12 | 8/28 | 9/23 | nt | 16/30 | 7/27 | 0/31 | 8/8 | 8/8 |
| **G22** | 0/12 | 0/12 | 8/8 | 8/8 | 2/9 | 14/14 | 5/13 | 12/21 | nt | 10/14 | 0/13 | 0/23 | 8/8 | 7/7 |
| **G23** | 0/4 | 0/12 | 5/5 | 5/5 | 3/18 | 25/25 | 9/20 | 16/19 | nt | 13/17 | 0/20 | 0/25 | 8/8 | 8/8 |
| **G24** | nt | nt | nt | nt | 3/12 | 17/17 | 7/13 | 7/9 | nt | 7/8 | 0/16 | 0/30 | 8/8 | 8/8 |
| **G25** | nt | nt | nt | nt | 5/24 | 25/25 | 4/25 | 12/25 | nt | 16/17 | 1/20 | 0/18 | 6/6 | 6/6 |
| **Total** | 0/215 | 0/200 | 168/168 | 171/171 | 129/431 | 313/452 | 61/533 | 119/453 | 23/297 | 196/518 | 62/510 | 42/624 | 195/195 | 182/182 |
| **%R** | 0% n=415 | | 100% n=339 | | 24.7% n=3818 | | | | | | | | 100% n=377 | |

**Supplementary file 1. Silencing capacities of *BX2^ON^* and *BX2^OFF^* lines throughout generations at 25°C and at 29°C.**
