# Supplementary material for: Environmentally-induced epigenetic conversion of a piRNA cluster
Source: eLife. 2019 Mar 15;8:e39842. doi: 10.7554/eLife.39842 (PMC6420265; doi:10.7554/eLife.39842)
Supplement: Supplementary file 2. — Five BX2, P(TARGET)GS lines showing full repression capacities after 23 generations kept at 29°C were transferred at 25°C and tested for their silencing capacities throughout generations. Numbers show females presenting full P(TARGET)GS repression and the total number of tested flies. In all cases, the BX2ON epiallele induced by high temperature remains completely stable during 50 additional generations at 25°C. [file elife-39842-supp2.docx]

| **Lines** | **1** | **2** | **3** | **4** | **5** |
| --- | --- | --- | --- | --- | --- |
| G1 | 4/4 | 3/3 | 4/4 | 3/3 | 4/4 |
| G6 | 2/2 | 4/4 | 4/4 | 4/4 | 5/5 |
| G7 | 7/7 | 8/8 | 8/8 | 6/6 | 8/8 |
| G10 | nt | 3/3 | nt | 5/5 | 5/5 |
| G11 | 6/6 | 6/6 | 6/6 | 6/6 | 6/6 |
| G14 | 2/2 | 8/8 | 8/8 | 1/1 | 3/3 |
| G16 | 5/5 | 5/5 | 6/6 | 6/6 | 6/6 |
| G25 | 3/3 | 6/6 | 6/6 | 8/8 | nt |
| G26 | 10/10 | 5/5 | 10/10 | 6/6 | 5/5 |
| G34 | 8/8 | 8/8 | 8/8 | 8/8 | 8/8 |
| G35 | 12/12 | 12/12 | 12/12 | 12/12 | 12/12 |
| G43 | 8/8 | 7/7 | 8/8 | 8/8 | 8/8 |
| G50 | 8/8 | 8/8 | 8/8 | 8/8 | 8/8 |
| **Total** | **75/75** | **83/83** | **88/88** | **81/81** | **78/78** |

**Supplementary file 2. Stability of *BX2^Θ^* lines.**
