# Supplementary material for: Environmentally-induced epigenetic conversion of a piRNA cluster
Source: eLife. 2019 Mar 15;8:e39842. doi: 10.7554/eLife.39842 (PMC6420265; doi:10.7554/eLife.39842)
Supplement: Supplementary file 3. — Reciprocal crosses were performed at 25°C between BX2ON, P(TARGET)GS (BX2Θ or BX2*) individuals and flies carrying a balancer of the second chromosome (Cy). For both the maternal and paternal inheritances (named MI and PI, respectively), lines were established and maintained at 25°C by crossing G1 individuals carrying the BX2Θ, P(TARGET)GS chromosome over Cy (Figure 1—figure supplement 2A). Silencing capacities of these lines was tested over generations by intra-strain ovarian ß-Galactosidase staining. Numbers represent the fraction of females showing complete repression of P(TARGET)GS. In all cases, maternal transmission of the BX2Θ cluster results in progeny showing complete repression capacities which are stable over generations whereas paternal transmission of the BX2Θ cluster results in the definitive loss of BX2 silencing capacities similarly to the BX2* epigenetic state. [file elife-39842-supp3.docx]

|  | *BX2^θ^* | | | | | | *BX2** | | | | | |
| --- | --- | --- | --- | --- | --- | --- | --- | --- | --- | --- | --- | --- |
| Lines | MI-1 | MI-2 | MI-3 | PI-1 | PI-2 | PI-3 | MI-1 | MI-2 | MI-3 | PI-1 | PI-2 | PI-3 |
| G1 | 7/7 | 8/8 | 8/8 | 0/8 | 0/8 | 0/8 | 3/3 | 5/5 | 8/8 | 0/5 | 0/5 | 0/6 |
| G2 | 8/8 | 8/8 | 8/8 | 0/8 | 0/8 | 0/8 | 8/8 | 8/8 | 8/8 | 0/8 | 0/8 | 0/12 |
| G3 | 8/8 | 7/7 | 8/8 | 0/8 | 0/8 | 0/8 | 8/8 | 8/8 | 8/8 | 0/8 | nt | 0/8 |
| G5 | 8/8 | 8/8 | 8/8 | 0/7 | 0/8 | 0/8 | 8/8 | 8/8 | 8/8 | 0/8 | 0/8 | 0/8 |
| G10 | 8/8 | 8/8 | 8/8 | 0/7 | 0/8 | 0/8 | 8/8 | 8/8 | 8/8 | 0/7 | 0/8 | 0/8 |
| G15 | 6/6 | 8/8 | 8/8 | 0/8 | 0/7 | 0/6 | 8/8 | 8/8 | 8/8 | 0/8 | 0/8 | 0/8 |
| G20 | 5/5 | 4/4 | 2/2 | 0/6 | 0/6 | 0/6 | 7/7 | 8/8 | 8/8 | 0/8 | 0/8 | 0/8 |
| Subtotal | 50/50 | 49/49 | 53/53 | 0/50 | 0/54 | 0/52 | 50/50 | 53/53 | 56/56 | 0/52 | 0/45 | 0/58 |
| Total |  | 152/152 |  |  | 0/156 |  |  | 159/159 |  |  | 0/155 |  |

**Supplementary file 3. Maternal effect of *BX2^ON^* lines.**
