# Supplementary material for: Environmentally-induced epigenetic conversion of a piRNA cluster
Source: eLife. 2019 Mar 15;8:e39842. doi: 10.7554/eLife.39842 (PMC6420265; doi:10.7554/eLife.39842)
Supplement: Supplementary file 4. — The capacity of the cytoplasm of BX2ON females to activate a BX2OFF cluster was tested as shown in the mating scheme. BX2ON females (either BX2Θ or BX2*) were crossed with BX2OFF males, incubated at 25°C. Lines were established with G1 individuals, which have maternally inherited piRNAs and paternally inheritance of the BX2 cluster (Figure 1—figure supplement 2B). These lines were maintained at 25°C and their silencing capacities were tested over generations by crossing females with P(TARGET)GS males. Numbers represent the fraction of females showing complete repression of P(TARGET)GS. All of the derived lines showed complete silencing capacities over generations, revealing that the cytoplasm of BX2ON females, either BX2Θ or BX2*, can fully activate a BX2OFF cluster. [file elife-39842-supp4.docx]

|  | *BX2** | | | *BX2^θ^* | | |
| --- | --- | --- | --- | --- | --- | --- |
| Lines | 1 | 2 | 3 | 1 | 2 | 3 |
| G1 | 8/8 | 8/8 | 10/10 | 10/10 | 9/9 | 9/9 |
| G2 | 10/10 | 10/10 | 10/10 | 10/10 | 10/10 | 10/10 |
| G3 | 8/8 | 8/8 | 8/8 | 8/8 | 8/8 | 7/7 |
| G5 | 6/6 | 7/7 | 7/7 | 8/8 | 5/5 | 7/7 |
| G10 | 6/6 | 3/3 | 6/6 | 6/6 | 6/6 | 6/6 |
| G15 | 8/8 | 8/8 | 7/7 | 7/7 | 8/8 | 6/6 |
| G20 | 4/4 | 5/5 | 5/5 | 7/7 | 8/8 | 4/4 |
| Subtotal | 50/50 | 49/49 | 53/53 | 56/56 | 54/54 | 49/49 |
| Total |  | 152/152 |  |  | 159/159 |  |

**Supplementary file 4. Paramutagenic effect of *BX2^ON^* lines.**
