# Supplementary material for: Environmentally-induced epigenetic conversion of a piRNA cluster
Source: eLife. 2019 Mar 15;8:e39842. doi: 10.7554/eLife.39842 (PMC6420265; doi:10.7554/eLife.39842)
Supplement: Supplementary file 6. — Same as Supplementary file 1 except that egg chambers were monitored for P(TARGET)GS repression instead of whole ovaries. Numbers show the fraction of repressed egg chamber per generation. [file elife-39842-supp6.docx]

|  | **25°C** | | | | **29°C** | | | | | | | | | |
| --- | --- | --- | --- | --- | --- | --- | --- | --- | --- | --- | --- | --- | --- | --- |
|  | ***BX2^OFF^*** | | ***BX2^ON^*** | | ***BX2^OFF^*** | | | | | | | | ***BX2^ON^*** | |
| Lines | **1** | **2** | **1** | **2** | **1A** | **1B** | **1C** | **1D** | **2A** | **2B** | **2C** | **2D** | **1** | **2** |
| **G1** | 0/840 | 0/980 | 960/960 | 960/960 | 52/1680 | 18/1680 | 33/1680 | 47/1800 | 26/1800 | 31/1800 | 36/1800 | 60/1800 | 1080/1080 | 1200/1200 |
| **G2** | 0/1320 | 0/1440 | 1440/1440 | 1440/1440 | 33/1080 | 11/1440 | 8/1200 | 102/1080 | 17/1560 | 4/840 | 112/1920 | 158/3360 | 1440/1440 | 1080/1080 |
| **G3** | 0/1440 | 0/360 | 840/840 | 960/960 | 145/2040 | 41/1680 | 42/1920 | 268/1680 | 155/2160 | 175/2760 | 444/2760 | 67/2760 | 840/840 | 1200/1200 |
| **G4** | 0/1800 | 0/1800 | 1200/1200 | 1200/1200 | 15/840 | 145/840 | 157/1920 | 251/1320 | 43/2040 | 156/2400 | 527/2760 | 195/2760 | 1200/1200 | 960/960 |
| **G5** | 0/1440 | 0/1440 | 1440/1440 | 1440/1440 | 278/2400 | 808/3720 | 405/4680 | 1362/240 | 67/4560 | 36/3960 | 72/360 | 39/2880 | 960/960 | 720/720 |
| **G6** | 0/720 | 0/960 | 960/960 | 960/960 | 75/240 | 524/2400 | 128/960 | 134/2160 | 67/1080 | 70/1800 | 214/1680 | 64/480 | 120/120 | 120/120 |
| **G7** | 0/960 | 0/960 | 960/960 | 960/960 | 750/2280 | 840/2520 | 318/4320 | 256/2520 | 249/4560 | 130/2400 | 422/2040 | 250/4440 | 840/840 | 960/960 |
| **G8** | 0/1200 | 0/960 | 720/720 | 960/960 | 425/3960 | 1352/3960 | 37/5880 | 1008/3720 | 614/2640 | 1684/4200 | 643/3960 | 743/6000 | 960/960 | 960/960 |
| **G9** | nt | nt | nt | nt | 1204/2760 | 1565/2520 | 243/2640 | 596/3360 | 361/3120 | 2093/5280 | 375/4320 | 514/4560 | 960/960 | 960/960 |
| **G10** | 0/960 | 0/960 | 960/960 | 960/960 | 970/2760 | 1920/2520 | 264/2640 | 492/2760 | 1324/2520 | 861/3000 | 854/4200 | 626/2880 | 960/960 | 960/960 |
| **G11** | 0/960 | 0/960 | 960/960 | 960/960 | 846/1680 | 1920/2040 | 214/1800 | 443/1800 | 120/1920 | 987/1920 | 352/1920 | 550/1920 | 960/960 | 960/960 |
| **G12** | 0/960 | 0/960 | 960/960 | 960/960 | 600/1680 | 840/840 | 130/600 | 9/960 | 9/720 | 244/2040 | 460/2160 | 641/2520 | 960/960 | 600/600 |
| **G13** | 0/1200 | 0/240 | 720/720 | 600/600 | 840/1320 | 1320/1320 | 132/4440 | 131/1080 | nt | 732/2280 | 161/3000 | 603/2160 | 1080/1080 | 960/960 |
| **G14** | 0/720 | 0/1200 | 600/600 | 600/600 | 3395/4920 | 3000/3000 | 20/2880 | 690/4920 | nt | 720/1320 | 33/2880 | 882/2880 | 720/720 | 600/600 |
| **G15** | 0/1440 | 0/480 | 960/960 | 960/960 | 1687/2640 | 1920/1920 | 367/2520 | 156/2760 | nt | 1440/2760 | 148/2880 | 244/2400 | 960/960 | 960/960 |
| **G16** | 0/1440 | 0/1320 | 960/960 | 600/600 | 360/1320 | 2040/2040 | 130/2040 | 265/2040 | nt | 1362/2400 | 263/2400 | 21/2280 | 960/960 | 960/960 |
| **G17** | 0/960 | 0/1440 | 600/600 | 840/840 | 480/1080 | 2640/2640 | 480/2160 | 101/720 | nt | 1128/1920 | 40/1560 | 21/2280 | 960/960 | 360/360 |
| **G18** | 0/1320 | 0/1440 | 960/960 | 960/960 | 249/960 | 720/720 | 124/1200 | 6/600 | nt | 1569/2520 | 365/960 | 1/1680 | 960/960 | 960/960 |
| **G19** | 0/1320 | 0/1440 | 720/720 | 960/960 | 750/5040 | 2520/2520 | 556/4560 | 727/2040 | nt | 1942/3840 | 616/2160 | 18/5280 | 960/960 | 960/960 |
| **G20** | 0/1440 | 0/360 | 720/720 | 360/360 | 484/1560 | 2520/2520 | 520/2040 | 1207/2160 | nt | 1366/2400 | 1460/3960 | 4/4320 | 960/960 | 960/960 |
| **G21** | 0/1440 | 0/1440 | 960/960 | 960/960 | 608/1920 | 1440/1440 | 1011/3360 | 1102/2760 | nt | 1922/3600 | 907/3240 | 6/3720 | 960/960 | 960/960 |
| **G22** | 0/1440 | 0/1440 | 960/960 | 960/960 | 259/1080 | 1680/1680 | 513/1560 | 1560/2520 | nt | 1200/1680 | 16/1560 | 11/2760 | 960/960 | 840/840 |
| **G23** | 0/480 | 0/1440 | 600/600 | 480/480 | 372/3160 | 3000/3000 | 1110/2400 | 1920/2280 | nt | 1571/2040 | 38/2400 | 15/3000 | 960/960 | 960/960 |
| **G24** | nt | nt | nt | nt | 361/1440 | 2040/2040 | 844/1560 | 842/1080 | nt | 844/960 | 44/1920 | 4/3600 | 960/960 | 960/960 |
| **G25** | nt | nt | nt | nt | 622/2880 | 3000/3000 | 525/3000 | 1448/3000 | nt | 1920/2040 | 163/2400 | 11/2160 | 720/720 | 720/720 |
| **Total** | 0/25800 | 0/24000 | 20160/20160 | 20040/20040 | 15860/51720 | 37824/54000 | 8311/63960 | 15123/54360 | 3052/28680 | 24187/62160 | 8765/61200 | 5748/74880 | 24600/24600 | 23040/23040 |

**Supplementary file 6. Silencing capacities of *BX2^ON^* and *BX2^OFF^* lines across generations cultured at 25°C and at 29°C.**
