# Supplementary material for: Environmentally-induced epigenetic conversion of a piRNA cluster
Source: eLife. 2019 Mar 15;8:e39842. doi: 10.7554/eLife.39842 (PMC6420265; doi:10.7554/eLife.39842)
Supplement: Supplementary file 7. — Small RNAs were prepared from ovaries of females of the indicated genotype. Values for the different categories of sequences are the total number of sequence reads that matched reference libraries. For comparisons, libraries were normalized (normalization factor) to 1 million miRNA (miRNA rpm) or to 1 million Dmel reads (Dmel rpm). [file elife-39842-supp7.docx]

| **Library ID** | **GRH160** | **GRH161** | **GRH162** | **GRH163** | **GRH164** | **GRH165** |
| --- | --- | --- | --- | --- | --- | --- |
| Genotype | *BX2^OFF^* | | | | | |
| Temperature | 25°C | | | 29°C | | |
| D. melanogaster Jan. 2013 (Flybase) | 3,288,5973 | 3,198,7242 | 3,135,9878 | 3,147,6903 | 364,50841 | 269,32299 |
| dme_miR_r20 | 8,098,219 | 7,458,369 | 7,225,648 | 6,804,381 | 8,472,003 | 6,374,911 |
| Dmel_all-tRNA | 549,423 | 676,558 | 587,138 | 475,249 | 793,839 | 410,800 |
| Dmel_all-miscRNA | 4,328,239 | 6,119,121 | 4,001,654 | 6,605,018 | 7,361,768 | 5,374,259 |
| Dmel_transposon_set_BDGP_v941 | 11,688,163 | 10,268,266 | 11,511,292 | 9,968,778 | 11,046,992 | 8,462,052 |
| D. melanogaster transcripts Jan. 2013 (Flybase r5.49) | 4,656,410 | 4,732,913 | 4,796,480 | 4,427,408 | 5,930,087 | 3,777,340 |
| Normalization factor miRNA rpm | 0.123 | 0.134 | 0.138 | 0.147 | 0.118 | 0.157 |
| **Normalization factor Dmel rpm** | **0.030** | **0.031** | **0.032** | **0.032** | **0.027** | **0.037** |

**Supplementary file 7. Annotation of small RNA libraries from *BX2^OFF^* raised at 25°C or 29°C.**
