# Supplementary material for: Environmentally-induced epigenetic conversion of a piRNA cluster
Source: eLife. 2019 Mar 15;8:e39842. doi: 10.7554/eLife.39842 (PMC6420265; doi:10.7554/eLife.39842)
Supplement: Supplementary file 8. — Comparison of the conversion frequency in one generation between BX2, P(TARGET)GS (Figure 3) and BX2 (Figure 3—figure supplement 1) genotypes. The difference between the presence and absence of the P(TARGET)GS transgene is highly significant (p=8.5×10^−6^, homogeneity χ2 = 23.35 with 2 degrees of freedom). [file elife-39842-supp8.docx]

|  | Number of G2 females | | |
| --- | --- | --- | --- |
| Repression occurrence | none | partial | complete |
| with *P(TARGET)^GS^* | 1789 | 24 | 17 |
| without *P(TARGET)^GS^* | 1136 | 1 | 0 |

**Supplementary file 8. *P(TARGET)^GS^* requirement in the *BX2* conversion process.**
