# Supplementary material for: Environmentally-induced epigenetic conversion of a piRNA cluster
Source: eLife. 2019 Mar 15;8:e39842. doi: 10.7554/eLife.39842 (PMC6420265; doi:10.7554/eLife.39842)
Supplement: Supplementary file 9. — BX2OFF was initially recombined with a line carrying the P(TARGET)GS transgene to obtain the BX2OFF, P(TARGET)GS lines. From these crosses, eight independent recombinants without the P(TARGET)GS transgene were recovered and were further cultured at 29°C. To test if some of them acquired silencing capacities, females were crossed with males harboring the P(TARGET)GS transgene and their progeny was stained for ß-Galactosidase expression. Numbers show the fraction of females harboring complete germline repression of P(TARGET)GS at each generation. A complete stability of the initial epigenetic OFF state was observed for all recombinant lines. [file elife-39842-supp9.docx]

|  | ***BX2^OFF^* recombined *in a P(TARGET)^GS^* genetic background** | | | | | | | | |
| --- | --- | --- | --- | --- | --- | --- | --- | --- | --- |
| **Lines** | #2 | #5 | #8 | #12 | #13 | #16 | #17 | #19 | **Total** |
| **G1** | 0/20 | 0/68 | 0/78 | 0/21 | 0/30 | 0/57 | 0/59 | 0/56 | 0/389 |
| **G5** | 0/82 | 0/50 | 0/115 | 0/86 | 0/107 | 0/86 | 0/60 | 0/87 | 0/673 |
| **G13** | 0/128 | 0/137 | 0/165 | 0/108 | 0/140 | 0/151 | 0/103 | 0/43 | 0/975 |
| **G30** | 0/27 | 0/37 | 0/25 | 0/24 | 0/22 | 0/51 | 0/33 | nt | 0/219 |
| **Total** | 0/257 | 0/292 | 0/383 | 0/239 | 0/299 | 0/345 | 0/255 | 0/186 | 0/2256 |

**Supplementary file 9. Silencing capacities of *BX2^OFF^* lines recombined in a *P(TARGET)^GS^* background throughout generations developed at 29°C.**
