# Supplementary material for: Environmentally-induced epigenetic conversion of a piRNA cluster
Source: eLife. 2019 Mar 15;8:e39842. doi: 10.7554/eLife.39842 (PMC6420265; doi:10.7554/eLife.39842)
Supplement: Supplementary file 10. — Numbers show the fraction of females harboring complete germline repression of P(TARGET)G at each generation. Complete stability of the initial epigenetic state was observed at 25°C for BX2OFF, 0% repression (n = 189). At 29°C, BX2OFF; P(TARGET)G lines showed emergence of silencing capacities, 19.79% (n = 6766). [file elife-39842-supp10.docx]

|  | 25°C | | 29°C | | | | | | |
| --- | --- | --- | --- | --- | --- | --- | --- | --- | --- |
| Lines | 1 | 2 | V4 | V6 | n6 | n7 | n8 | n9 | n10 |
| G1 |  |  | 0/92 | 0/16 | 0/51 | 0/42 | 0/128 | 5/58 | 0/108 |
| G2 | 0/22 | 0/32 | 0/106 | 0/32 | 0/221 | 0/117 | 3/261 | 30/153 | 0/269 |
| G3 |  |  | 0/86 | 0/21 | 0/150 | 1/167 | 5/185 | 61/160 | 0/208 |
| G4 |  |  | 0/45 | 13/41 | 0/138 | 0/146 | 19/161 | 43/88 | 0/142 |
| G5 |  |  | 1/37 | 25/56 | 0/106 | 0/101 | 16/83 | 40/83 | 0/82 |
| G6 |  |  | 1/17 | 49/73 | 0/111 | 0/108 | 16/89 | 65/95 | 0/74 |
| G7 |  |  | 2/9 | 111/151 | 0/85 | 0/75 | 0/92 | 76/130 | 0/29 |
| G8 | 0/22 | 0/15 | 3/24 |  |  |  |  |  |  |
| G9 |  |  | 9/12 |  |  |  |  |  |  |
| G10 |  |  | 0/23 | 38/63 |  |  |  |  |  |
| G11 |  |  | 1/3 | 69/106 |  |  |  |  |  |
| G12 | 0/20 | 0/23 | 2/25 | 44/67 |  |  |  |  |  |
| G13 |  |  | 8/75 | 139/200 |  |  |  |  |  |
| G14 |  |  | 9/116 |  |  |  |  |  |  |
| G15 |  |  |  | 141/193 |  |  |  |  |  |
| G16 |  |  |  |  |  |  |  |  |  |
| G17 |  |  | 43/130 |  |  |  |  |  |  |
| G18 |  |  | 83/249 |  |  |  |  |  |  |
| G19 |  |  | 119/284 |  |  |  |  |  |  |
| G20 | 0/14 | 0/14 | 49/118 |  |  |  |  |  |  |
| G21 |  |  |  |  |  |  |  |  |  |
| G22 |  |  |  |  |  |  |  |  |  |
| G23 |  |  |  |  |  |  |  |  |  |
| G24 | 0/7 | 0/13 |  |  |  |  |  |  |  |
|  |  |  |  |  |  |  |  |  |  |
| Total | 0/92 | 0/97 | 330/1451 | 629/1019 | 0/862 | 1/756 | 59/999 | 320/767 | 0/912 |
| %R | 0% n=189 | | 19.79% n=6766 | | | | | | |

**Supplementary file 10. Silencing capacities of *BX2^OFF^; P(TARGET)^G^* lines throughout generations at 25°C and at 29°C.**
