# Supplementary material for: Environmentally-induced epigenetic conversion of a piRNA cluster
Source: eLife. 2019 Mar 15;8:e39842. doi: 10.7554/eLife.39842 (PMC6420265; doi:10.7554/eLife.39842)
Supplement: Supplementary file 11. — BX2OFF, P(TARGET)GS flies were raised during one generation either on classical cornmeal medium (control) at 25°C or were heat shocked for 1 hr at 37°C at the 0–2 hr embryo stage or were cultured on medium supplemented with 150 mM NaCl. The female progeny were then stained for ß-Galactosidase expression and egg chambers were individually monitored in order to detect any possible conversion event. No repressed egg chambers were observed (0/total number of egg chambers). Compared to results obtained at 29°C (from data observed in G1 in Figure 3), differences are significant: for heat shock experiment, p=7.2×10^−25^, homogeneity χ2 = 106.03 with 2 degrees of freedom and for NaCl experiment, p=4.9×10^−27^, homogeneity χ2 = 115.93 with 2 degrees of freedom. [file elife-39842-supp11.docx]

|  | 29°C | Control 25°C | + Heat Shock | + NaCl |
| --- | --- | --- | --- | --- |
| *BX2^OFF^, P(TARGET)^GS^* | 586/≈21720 | 0/≈1680 | 0/≈3840 | 0/≈4200 |

**Supplementary file 11. Heat shock and saline stresses do not induce conversion of *BX2^OFF^*.**
