# Supplementary material for: Environmentally-induced epigenetic conversion of a piRNA cluster
Source: eLife. 2019 Mar 15;8:e39842. doi: 10.7554/eLife.39842 (PMC6420265; doi:10.7554/eLife.39842)
Supplement: Transparent reporting form [file elife-39842-transrepform.docx]

***eLife’s* transparent reporting form**

We encourage authors to provide detailed information *within their submission* to facilitate the interpretation and replication of experiments. Authors can upload supporting documentation to indicate the use of appropriate reporting guidelines for health-related research (see [EQUATOR Network](http://www.equator-network.org/%20)), life science research (see the [BioSharing Information Resource](https://biosharing.org/)), or the [ARRIVE guidelines](http://www.plosbiology.org/article/info:doi/10.1371/journal.pbio.1000412) for reporting work involving animal research. Where applicable, authors should refer to any relevant reporting standards documents in this form.

If you have any questions, please consult our Journal Policies and/or contact us: [editorial@elifesciences.org](mailto:editorial@elifesciences.org).

**Sample-size estimation**

- You should state whether an appropriate sample size was computed when the study was being designed
- You should state the statistical method of sample size computation and any required assumptions
- If no explicit power analysis was used, you should describe how you decided what sample (replicate) size (number) to use

Please outline where this information can be found within the submission (e.g., sections or figure legends), or explain why this information doesn’t apply to your submission:

All sample sizes of females tested were chosen as large as possible and are provided in each experiments in Figures, Figure supplements and Supplementary files.

RT-qPCR and ChIP-qPCR were classically performed using a sample size of 6 and 5 respectively.

**Replicates**

- You should report how often each experiment was performed
- You should include a definition of biological versus technical replication
- The data obtained should be provided and sufficient information should be provided to indicate the number of independent biological and/or technical replicates
- If you encountered any outliers, you should describe how these were handled
- Criteria for exclusion/inclusion of data should be clearly stated
- High-throughput sequence data should be uploaded before submission, with a private link for reviewers provided (these are available from both GEO and ArrayExpress)

Please outline where this information can be found within the submission (e.g., sections or figure legends), or explain why this information doesn’t apply to your submission:

We considered Biological replicate when multiple samples are obtained from different extraction experiments, and technical replicates when the same sample is tested multiple times.

The epigenetic conversion of *BX2* locus induced by high temperature was obtained in six independent set of experiments. The two last ones are presented in the manuscript because they were performed on bigger size sample.

In the experiment presented in the manuscript, the biological samples were indicated as lines in Supplementary files 1, 2, 3, 4, 6, 9 and 10. The number of females tested at each generation are indicated in the respective files.

For the RT qPCR, up to 6 biological replicates were used for each genotype with 3 technical samples (Figure 4A, B, D and Figure 4 - figure supplement 1.

For the ChIP qPCR presented in Figure 2D-E, 5 biological replicates were tested.

The small RNA sequences were performed in one sample composed of 20 pairs of ovaries for the Figure 2A, B, C and Figure 2 - figure supplement 1 and in triplicate in Figure 4 E, F, G, H, I and J. The sequences have been deposited at the GEO under accession number GSE116122. This information can be found in the Materials and Methods section.

**Statistical reporting**

- Statistical analysis methods should be described and justified
- Raw data should be presented in figures whenever informative to do so (typically when N per group is less than 10)
- For each experiment, you should identify the statistical tests used, exact values of N, definitions of center, methods of multiple test correction, and dispersion and precision measures (e.g., mean, median, SD, SEM, confidence intervals; and, for the major substantive results, a measure of effect size (e.g., Pearson's r, Cohen's d)
- Report exact p-values wherever possible alongside the summary statistics and 95% confidence intervals. These should be reported for all key questions and not only when the p-value is less than 0.05.

Please outline where this information can be found within the submission (e.g., sections or figure legends), or explain why this information doesn’t apply to your submission:

ChIP qPCR : An unpaired t-test was used to calculate significance of the differences (p < 0.05). Error bars represent the Standard Deviation.

For RT-qPCR, the bilateral Student's t-test was applied to compare the effects between 25 and 29°C on *BX2* conversion.

Homogeneity χ^2^ tests were used to test the effect of different euchromatic homologous sequences in *BX2* heat conversion and are shown in text and in Supplementary file 8.

(For large datasets, or papers with a very large number of statistical tests, you may upload a single table file with tests, Ns, etc., with reference to sections in the manuscript.)

**Group allocation**

- Indicate how samples were allocated into experimental groups (in the case of clinical studies, please specify allocation to treatment method); if randomization was used, please also state if restricted randomization was applied
- Indicate if masking was used during group allocation, data collection and/or data analysis

Please outline where this information can be found within the submission (e.g., sections or figure legends), or explain why this information doesn’t apply to your submission:

No grouping was applied.

**Additional data files (“source data”)**

- We encourage you to upload relevant additional data files, such as numerical data that are represented as a graph in a figure, or as a summary table
- Where provided, these should be in the most useful format, and they can be uploaded as “Source data” files linked to a main figure or table
- Include model definition files including the full list of parameters used
- Include code used for data analysis (e.g., R, MatLab)
- Avoid stating that data files are “available upon request”

Please indicate the figures or tables for which source data files have been provided:

Additional data files can be found in the manuscript including:

-Map and name of the transgenes used in this study to see common homologous regions (Figure 1, figure supplement 1A-B)

-Schematics of crosses to a better comprehension of the experiment design (Figure 1, figure supplement 2)

-analysis of small RNA at the *42AB* classical piRNA cluster to show that temperature has no effect on an already active piRNA cluster (Figure 2, figure supplement 1).

-mapping of small RNA at the *AGO1* locus to see that this locus is not a natural piRNA producing locus (Figure 2, figure supplement 2)

-schematic representation of the drosophila chromosome highlighting that only the *BX2* locus is producing new piRNA after heat treatment (Figure 2, figure supplement 3).

- testing hypothesis of a low conversion rate per generation can explain the delay of the *BX2Ɵ* occurrence. A theoretical model is proposed and compare to the observed data (Figure 1, figure supplement 3)

-qRTPCR measurements of *AGO1* in different genetic background to confirm the effect of high temperature on *AGO1* RNA amount (Figure 4, figure supplement 1)

- schematic of the experiment of heat treatment on the *BX2* locus without the *P(TARGET*)*^GS^* reporter transgene (Figure 3, figure supplement 1)

- schematic of the experiment of heat treatment on the *BX2* locus with another *P(TARGET)^GS^* reporter transgene not expressed in the germline (Figure 3, figure supplement 2)

- *BX2* paramutated cluster is a genuine germline piRNA cluster showed by its requirement of *Moonshiner* a recently identified transcription factor for piRNA cluster (Figure 3, figure supplement 3).

- Complementary RNA steady-state measurements by quantitative RT-PCR (Figure 4, figure supplement 1).
